# Valeur diagnostique de la tomodensitométrie dans la cysticercose cérébrale à Lomé

**DOI:** 10.11604/pamj.2015.20.67.6085

**Published:** 2015-01-26

**Authors:** Lantam Sonhaye, Mazamaesso Tchaou, Abdoulatif Amadou, Kouméabalo Assih, Berésa Kolou, Komlavi Adjenou, Koffi N'dakena

**Affiliations:** 1 Service de Radiologie CHU Lomé, Lomé, Togo

**Keywords:** Neurocyscitercose, calcification cérébrale, épilepsie, tomodensitométrie, neurocysticercosis, cerebral calcification, epilepsy, TDM

## Abstract

La cysticercose a une prévalence élevée dans les pays sub-sahariens et son diagnostic reste difficile. Cette étude a pour but de déterminer la valeur diagnostique de la tomodensitométrie dans la cysticercose cérébrale. Recrutement des patients ayant bénéficié d'une tomodensitométrie cérébrale dans l'une des circonstances suivantes: épilepsie, hypertension intracrânienne, syndrome pyramidal, détérioration mentale, ataxie locomotrice ou une diminution de l'acuité visuelle. Les critères de Del Brutto et al. avaient permis de retenir le diagnostic positif de cysticercose cérébrale. Pendant la période, 4193 patients avaient été inclus à l’étude, dont 140 cas de cysticercose cérébrale (3,3%). L’âge moyen des patients de cysticercose cérébrale était de 36 ± 14 ans, avec des extrêmes de 17 ans et 59 ans. La sensibilité et la spécificité de la tomodensitométrie dans la cysticercose cérébrale sont respectivement de 96,4% et 98,3%. La valeur prédictive positive et la valeur prédictive négative de la tomodensitométrie sont respectivement de 65,9% et 99,8%. Les aspects tomodensitométriques chez les vrais positifs sont dominés par des lésions associées, 72 cas (53,3%), suivies d'une hypodensité nodulaire arrondie unique ou multiple sans prise de contraste iodée, 17cas (12,6%). La TDM est une technique d'imagerie qui a une sensibilité et une spécificité élevées dans le diagnostic de la cysticercose cérébrale. Cependant, les autres critères de diagnostic restent utiles du fait de l'existence de nombreux cas de faux positifs à la tomodensitométrie.

## Introduction

La cysticercose est une cestodose larvaire causée par le parasite *Taenia solium*; elle est liée à l'ingestion par l'Homme ou le porc des œufs du parasite présents dans l'eau ou l'alimentation souillée par les déjections humaines. Ainsi, ces œufs libéreront des embryons capables de s'enkyster dans différents organes, les localisations les plus redoutées étant l’œil et le système nerveux central (neurocysticercose) [1]. La cysticercose est une zoonose cosmopolite et endémique dans de nombreux pays. Les foyers endémiques reconnus par l'Organisation Mondiale de la Santé (OMS) sont l'Amérique Centrale et du Sud, l'Asie et l'Afrique (Afrique Sub-Saharienne) [1, 2]. Comme dans la plupart des pays en développement, la prévalence de la cysticercose est sous-estimée en Afrique en raison d'un manque d'infrastructures médicales et de diagnostic appropriés [3]. Toutefois, la cysticercose est retrouvée en Afrique du Sud, au Bénin, au Burundi, au Cameroun, en Côte d'Ivoire, à Madagascar, au Sénégal, au Togo, au Zimbabwe et à l'Ile de la Réunion avec une prévalence estimée entre 0,45 et 30% [4]. Le diagnostic de la cysticercose n'est pas aisé en raison de ces manifestations polymorphes. Il est basé sur l'association de données cliniques, épidémiologiques, radiologiques et immunologiques [5]. L'imagerie médicale joue un rôle prépondérant dans le diagnostic de la cysticercose et plus particulièrement pour la cysticercose cérébrale (CC) [6]. Malgré la forte prévalence de cette maladie dans les pays sub-sahariens, aucune étude n'a été faite sur la valeur diagnostique de la tomodensitométrie dans la cysticercose cérébrale. Ainsi, cette étude a pour but de déterminer la valeur diagnostique et de de décrire les aspects tomodensitométriques dans la cysticercose cérébrale.

## Méthodes

Nous avons recruté entre le 1^er^ janvier 2011 et le 31 décembre 2013 (soit une période de 36 mois) au service de radiologie du CHU Campus de Lomé, tout patient ayant bénéficié d'une tomodensitométrie (TDM) cérébrale pour l'une des indications suivantes: épilepsie, hypertension intracrânienne, syndrome pyramidal, détérioration mentale, ataxie locomotrice, ou une diminution de l'acuité visuelle. Le diagnostic avait été posé sur la base d'arguments cliniques, épidémiologiques, biologiques et radiologiques. Nous avons exclus de l’étude, les patients dont le dossier médical était incomplet pouvant permettre de retenir un diagnostic. L'examen TDM était réalisé sans et avec injection de produit de contraste iodé, sur un appareil de marque General Electric 16 barrettes; l'analyse des clichés était faite en fenêtre parenchymateuse sur des coupes fines en reconstructions axiales, parfois sagittales et coronales. Les paramètres étudiés sont: l’âge, le sexe et les aspects de la TDM. On avait calculé la sensibilité et la spécificité en en définissant les cas de neurocysticercose selon les critères proposés par Del Brutto et al.: Présence d'un critère absolu et d'un critère épidémiologique, ou de deux critères majeurs associés à un critère mineur et d'un critère épidémiologique (Tableau 1) [7]. On parlera: de vrai positif (VP), lorsque la TDM avait évoqué la CC et que les autres critères l'avaient confirmées; de faux positif (FP), lorsque la TDM avait évoqué la CC et que les autres critères n’étaient retrouvés; de faux négatifs (FN), lorsque la TDM était négative et que le diagnostic de la CC était retenu sur la base des autres critères; de vrais négatifs (VN), lorsque la TDM était négative et que le diagnostic de la CC n’était pas retenu.

**Tableau 1:** Critères diagnostiques de la neurocysticercose [7]

Critères	Eléments du diagnostic
**Absolus**	démonstration histologique du parasite dans une biopsie lésions kystiques comportant un scolex visible à la TDM ou à l'imagerie par résonance magnétique (IRM) visualisation directe de parasites sous-rétiniens au fond d’æil
**Majeurs**	lésions kystiques suggestives à la TDM ou à l'IRM examen sérologique positif résolution des lésions cérébrales sous traitement antiparasitaire
**Critères mineurs**	lésions compatibles à la TDM ou à l'IRM tableau clinique suggestif examen du liquide céphalo-rachidien positif cysticercose confirmée en dehors du système nerveux central
**Epidémiologiques**	contact avec un individu infecté individus provenant d'une zone d'endémie voyage fréquent dans une zone d'endémie

## Résultats

**Figure 1:**
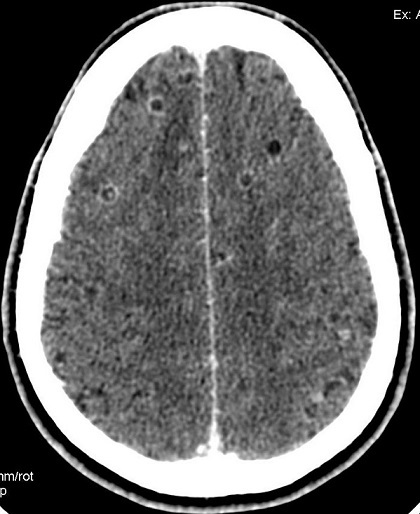
Coupe TDM axiale avec injection de produit de contraste mettant en évidence des hypodensités arrondies, bien limitées avec un rehaussement des parois, sans prise de contraste des parois et avec une hyperdensité central

**Figure 2:**
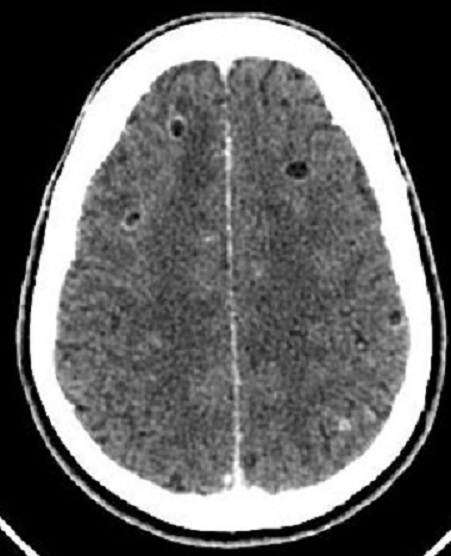
Coupe TDM axiale avec injection de produit de contraste mettant en évidence des hypodensités arrondies, bien limitées avec un rehaussement des parois

**Figure 3:**
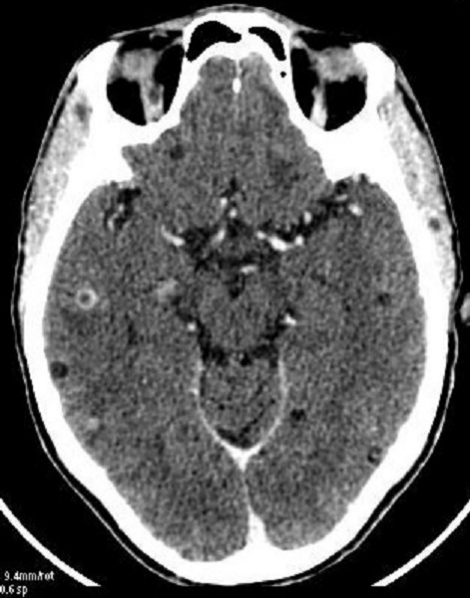
Coupe TDM axiale avec injection de produit de contraste mettant en évidence des hypodensités multifocales frontales, des hypodensités arrondies, bien limitées avec un rehaussement des parois, sans prise de contraste des parois et avec une hyperdensité centrale traduisant le scolex

Pendant la période, 4193 patients avaient été inclus à l’étude, dont 140 cas de CC, soit une fréquence de la CC de 3,3%. L’âge moyen des patients ayant une cysticercose était de 36 ± 14 ans, avec des extrêmes de 17 ans et 59 ans (Tableau 2). On avait noté une prédominance masculine avec un sex ratio de 3,5 (109hommes pour 31femmes). Les vrais positifs représentaient 135cas, les faux positifs 70 cas, les vrais négatifs 3983cas, et les faux négatifs 05cas. La sensibilité (Se) et la spécificité (Sp) de la tomodensitométrie dans la cysticercose cérébrale sont respectivement de 96,4% et 98,3%. La valeur prédictive positive (VPP) et la valeur prédictive négative (VPN) de la tomodensitométrie dans la cysticercose cérébrale sont respectivement de 65,9% et 99,8%. Chez les cinq cas de faux négatifs de cysticercose cérébrale, l'examen TDM était normal. Les aspects tomodensitométriques chez les vrais positifs (Tableau 3) sont dominés par des lésions associées, 72cas (53,3%), suivies d'une hypodensité nodulaire arrondie unique ou multiple sans prise de contraste iodée (Figure 1), 17cas (12,6%); on avait noté 14 cas (10,4%) d'hypodensité nodulaire arrondie unique ou multiple de 5 à 20mm avec une hyperdensité centrale (Figure 1). Les hypodensités nodulaires arrondies uniques ou multiples avec une prise de contraste iodée annulaire (Figure 2) représentaient 12 cas (08,9%), et les hypodensités parenchymateuses multifocales (Figure 3) 2 cas (01,5%). Les aspects tomodensitométriques chez les faux positifs (Tableau 4) sont dominés par une hypodensité nodulaire arrondie unique ou multiple sans prise de contraste iodée, 17 cas (24,3%).

**Tableau 2:** Répartition des patients de cysticercose cérébrale selon l’âge

	Avant 10 ans	[10-20[	[20-30[	[30-40[	[40-50[	50 ans et plus	Total
Nombre	00	7	24	30	37	42	140
Pourcentage	00	05,0	17,2	21,4	26,4	30,0	100

**Tableau 3:** Répartition des patients selon les aspects tomodensitométriques de la cysticercose cérébrale chez les vrais positifs

Aspects TDM	Nombre (N = 135)	Pourcentage (%)
**Lésions isolées**	**63**	**46,7**
Hypodensité nodulaire arrondie unique ou multiple de 5 à 20mm (N1)	17	12,6
Image nodulaire calcifiée unique ou multiple (N4)	17	12,6
Hypodensité nodulaire arrondie unique ou multiple de 5 à 20mm avec une hyperdensité punctiforme centrale (N2)	14	10,4
Hypodensité nodulaire arrondie unique ou multiple de 5 à 20mm, avec un annulaire (N3)	12	08,9
Hypodensité multifocale ou diffuse sans prise de contraste iodée(H1)	02	01,5
Hypodensité multifocale ou diffuse avec prise de contraste iodée (H2)	01	00,7
**Lésions associées**	**72**	**53,3**
N1+ N3 (Figure 2)	24	17,7
N1 + N2 + N3 (Figure 1)	17	12,6
N1 + N3 + N4	10	07,4
N2 + N3 + N4	10	07,4
H1+ N1 + N2 + N3 (Figure 3)	07	05,2
H1 + H2	02	01,5
H1 + N1 + N2	02	01,5
**Total**	**135**	**100**

**Tableau 4:** Répartition des patients selon les aspects tomodensitométriques chez les faux positifs

Aspects TDM	Nombre (N = 70)	Pourcentage (%)
Hypodensité multifocale ou diffuse sans prise de contraste iodée(H1)	00	00
Hypodensité multifocale ou diffuse avec prise de contraste iodée(H2)	00	00
Hypodensité nodulaire arrondie unique ou multiple de 5 à 20mm (N1)	17	24,3
Hypodensité nodulaire arrondie unique ou multiple de 5 à 20mm avec une hyperdensité punctiforme centrale (N2)	00	00
Hypodensité nodulaire arrondie unique ou multiple de 5 à 20mm, avec un rehaussement des bords hyperdenses et hypodensité péri-lésionnelle (N3)	14	20,0
Image nodulaire calcifiée unique ou multiple (N4)	14	20,0
N1 + N3	11	15,7
N1 + N4	11	15,7
N3 + N4	3	04,3
**Total**	**70**	**100**

## Discussion

La cysticercose a une prévalence estimée entre 0,45 et 30% dans certains pays africains sub-sahariens [4]. La fréquence de la cysticercose cérébrale dans notre étude est de 3,3%. Cette fréquence pourrait mal refléter la prévalence actuelle de la cysticercose, ceci du fait de l'existence des formes asymptomatiques de la maladie. Selon les données de la littérature,la cysticercose est une zoonose présente à tous les âges affectant préférentiellement l'adulte plutôt que l'enfant et dont la prévalence à tendance à augmenter avec l’âge [8–11]. La période d'incubation plus ou moins longue, un diagnostic difficile et, l'effet cumulatif de l'exposition avec l’âge lié aux activités pratiquées peuvent expliquer cette différence [10]. Dans notre étude l’âge moyen des patients étaient de 36 ± 14 ans, avec des extrêmes de 17 ans et 59 ans, et le nombre de cas augmentait avec l’âge (Tableau 2). Il n'a pas été démontré de différence significative de prévalence liée au sexe. Cependant, il semblerait que la réaction inflammatoire soit plus intense chez les femmes et probablement sous contrôle hormonal et qu'elles présentent un nombre de parasites calcifiés plus important avec une réaction immunitaire semble-t-il plus efficace chez la femme que chez l'homme [10]. Une prédominance masculine s'est dégagée dans notre étude avec un sexe ration de 3,5 (109 hommes contre 31 femmes). Dans la littérature, plusieurs critères sont utilisés pour le diagnostic de la cysticercose;Ces formes sont classées stade I pour Duchene et al. [12]. Dans notre étude, les critères modifiés de Del Brutto ont été utilisés [7]. La tomodensitométrie a une sensibilité élevée dans le diagnostic de la cysticercose cérébrale. Pour Palacio et al. [13], la sensibilité de la TDM est de 94,3%; elle est un peu plus élevée (95%) pour Garcia et al. [6]. Dans notre étude la sensibilité de la TDM est de 96,4%. Dans la littérature, la tomodensitométrie a une spécificité élevée dans le diagnostic de la cysticercose cérébrale; elle est supérieure à 95% pour Garcia et al. [6]. Dans notre étude la spécificité de la TDM est de 98,3%. Les valeurs prédictives négative et positive de la tomodensitométrie dans la cysticercose cérébrale sont rarement retrouvées dans la littérature. Dans notre étude, la valeur prédictive négative est de 99,8% et la valeur prédictive positive de 65,9%.

Dans le système nerveux central, la larve peut se localiser au niveau du parenchyme cérébral, des espaces sous-arachnoïdiens, à l'intérieur du système ventriculaire et plus rarement, au niveau de la moelle et du rachis [12]. Les localisations parenchymateuse et sous-arachnoïdienne sont fréquentes [14]. Dans notre étude, seule la forme parenchymateuse a été retrouvée. L'imagerie par résonance magnétique améliore le diagnostic des formes intra-ventriculaires et sous- arachnoïdiennes dans la littérature [15, 16]. La cysticercose cérébrale peut se présenter sous plusieurs aspects à la TDM. L'examen TDM peut même être normal; dans notre étude, 05 cas (3,6%) de faux négatifs avaient été retrouvés, avec un examen TDM normal. Il s'agit là des principales indications de l'IRM à la recherche d'une forme intra-ventriculaire et parfois intra-parenchymateuse [17]. Il s'agit des formes classées stade I pour Duchene et al. [12]. L'examen TDM peut montrer une association de lésions ou des lésions isolées. Les lésions associées constituent l'aspect tomodensitométrique le plus retrouvé [18]. Dans notre étude les lésions associées représentent 53,3% (72 cas) chez les vrais positifs. Ceci s'explique par le fait que plusieurs stades d’évolution de la maladie sont souvent retrouvés chez le même malade dans les pays à forte prévalence [19]. Les lésions isolées à la TDM sont multiples, mais dominées par des hypodensités nodulaires arrondies uniques ou multiples de 5 à 20 mm de diamètre, avec ou sans prise de contraste, et par des formations nodulaires calcifiées; les hypodensités nodulaires arrondies uniques ou multiples avec une hyperdensité centrale représentant le scolex sont beaucoup plus rares. Il s'agit de la mise en évidence du scolex, lésion pathogmonique de la maladie [16]. Dans notre étude, les hypodensités nodulaires arrondies uniques ou multiples avec une hyperdensité centrale avaient été retrouvées de façon isolée chez 14 cas (10,4% des vrais positifs). Les lésions nodulaires calcifiées uniques ou multiples isolées sont retrouvées dans 12,6% (17 cas) des vrais positifs. Il s'agit de l'aspect tomodensitométrique le plus fréquent dans la neurocysticercose asymptomatique [16, 20]. L'absence de spécificité de certains aspects tomodensitométriques expliquerait les faux positifs à la TDM. Dans notre série, 70 cas de faux positifs avaient été retrouvés. Et, leurs aspects tomodensitométriques sont dominés par hypodensités nodulaires arrondies uniques ou multiples de 5 à 20 mm (17 cas soit 24,3%). Il s'agit des lésions qui peuvent être rencontrées dans la tuberculose cérébrale, dans la toxoplasmose cérébrale ou dans les métastases cérébrales [16].

## Conclusion

La neurocysticercose est une maladie à forte prévalence dans les pays en développement. Son diagnostic nécessite des explorations radiologiques. La TDM est une technique d'imagerie de plus en plus disponible de nos jours et qui a une sensibilité (96,4%) et une spécificité (98,3%) élevées dans le diagnostic de la cysticercose cérébrale. Cependant, il est toujours nécessaire d'associer au diagnostic de cette maladie des critères épidémiologiques et biologiques du fait de l'existence de nombreux cas de faux positifs à la TDM qui a une valeur prédictive positive de 65,9%.

## References

[1] Roman G, Sotelo J, Del Brutto O, Flisser A, Aumas M, Wadia N, Botero D, Cruz M, Garcia H, De Bittencourt PMR, Trelles L, Arriagada C, Lorenzana P, Nash TE, Spina-Franca A (2000). A proposal to declareneurocysticercosis an international reportable disease. Bull WHO..

[2] Sciutto E, Fragoso G, Fleury A, Laclette JP, Sotelo J, Aluja A, Vargas L, Larralde C (2000). Taeniasoliumdisease in humans and pigs: an ancient parasitosis disease rooted in developing countries and emerging as a major health problem of global dimensions. Microbes Infect..

[3] Nguekam JP, Zoli AP, Zogo PO, Kamga AC, Speybroeck N, Dorny P, Brandt J, Losson B, Geerts S (2003). A seroepidemiological study of human cysticercosis in west Cameroon. Trop Med Int Health..

[4] Avode DG (1996). Epidémiologie de la neurocysticercose en Afrique Noire, Med d. Afr Noire..

[5] Hawk MW, Shahlaie K, Kim KD, Theis JH (2005). Neurocysticercosis: a review. SurgNeurol..

[6] Garcia HH, Del Brutto OH (2003). Imaging findings in neurocysticercosis. Acta Trop..

[7] Del Brutto OH, Rajshekhar V, White AC, Tsang VC, Nash TE, Takayanagui OM, Schantz PM, Evans CA, Flisser A, Correa D, Botero D, Allan JC, Sarti E, Gonzalez AE, Gilman RH, Garcia HH (2001). Proposed diagnostic criteria for neurocysticercosis. Neurology..

[8] Garcia HH, Pretell EJ, Gilmann RH, Martinez SM, Moulton LH, Del Brutto OH, Herrera G, Evans CA, Gonzalez AE (2004). A trial of antiparasitic treatment to reduce the rate of seizures due to cerebral cysticercosis. N Engl J Med..

[9] Salim L, Ang A, Handali S, Tsang VCW (2009). Seroepidemiologic survey of cysticercosis-taeniasis in four central highland districts of Papua, Indonesia. Am JTrop Med Hyg..

[10] Fleury A, Dessein A, Dumas M, Preux PM, Tapia G, Larralde C (2004). Symptomatic human neurocysticercosis: age, sex and exposure factors relating with diseaseheterogeneity. J Neurology..

[11] Fleury A, Morales J, Bobes RJ, Dumas M, Yanez O, Pina J (2006). An epidemiological study of familial neurocysticercosis in an endemic Mexican community. Trans R Soc Trop Med Hyg..

[12] Duchene M, Benoudiba F, Iffenecker C, Hadj-Rabia M, Caldas JGMP, Doyon D (1999). La neurocysticercose. J Radiol..

[13] Palacio G, Tobón ME, Mora O, Sánchez JL, Jiménez M, Muñoz A, Pineda D, Villa A, Londoño A, Buriticá O, Díaz H, Acebedo S, Giraldo M, Canasteros I, Tobón N, Gómez ME, Arana A, Uribe CS, Tsang V, Pilcher J, Ahn L, Rodríguez M, Hurtado A, Ceballos F, Jiménez I (1997). Prevalence of neurocysticercosis in individuals affected by epilepsy. Rev Neurol..

[14] Agapejev S (2011). Neurocysticercosis: the enigmatic disease. Cent NervSyst Agents Med Chem..

[15] Garcia HH, Del Brutto OH, Nash TE, White AC, Tsang VC, Gilman RH (2005). New concepts in the diagnosis and management of neurocysticercosis (Taeniasolium). Am J Trop Med Hyg..

[16] KRAFT R (2007). Cysticercosis: an emerging parasitic disease. Am Fam Physician.

[17] Sarria Estrada S, FrascheriVerzelli L, SiuranaMontilva S, Auger Acosta C, RoviraCañellas A (2013). Imaging findings in neurocysticercosis. Radiologia..

[18] Palacio LG, Jiménez I, Garcia HH, Jiménez ME, Sánchez JL, Noh J, Ahn L, Mora O, Giraldo M, Tsang VC (1998). Neurocysticercosis in persons with epilepsy in Medellín, Colombia. Epilepsia..

[19] Carpio A (2002). Neurocysticercosis: an update. Lancet Infect Dis..

[20] Roy B, Verma S, Awasthi R, Rathore RK, Venkatesan R, Yoganathan SA, Das JK, Prasad KN, Gupta RK (2011). Correlation of phase values with CT Hounsfield and R2* values in calcified neurocysticercosis. J MagnReson Imaging..

